# Thymofibrolipoma: a case report and review of the literature

**DOI:** 10.1186/s13000-022-01260-1

**Published:** 2022-10-12

**Authors:** Ryu Jokoji, Emiko Tomita

**Affiliations:** 1 Department of Pathology, Nippon Life Hospital, 2-1-54 Enokojima, Nishi-ku, 550-0006 Osaka, Japan; 2 Department of Thoracic Surgery, Nippon Life Hospital, Osaka, Japan

**Keywords:** Case report, Thymofibrolipoma, Lipofibroadenoma, Thymus

## Abstract

**Background:**

Thymofibrolipoma has been described as a variant of thymolipoma. To date, 3 cases have been reported, and the lesion have been described to consist of extensive areas of collagenous tissue interspersed with islands of mature adipose tissue and strands of thymic tissue.

**Case presentation:**

A 43-year-old woman had an anterior mediastinal tumor. Macroscopically, the cut surface of the tumor was composed of a yellowish lipomatous component and a uniform whitish fibrous component with elastic stiffness. Microscopically, the tumor was composed of collagenous fibrous tissue with sparse spindle cells, mature adipocytes and strands or islands of thymic tissue. The spindle cells in the fibrous tissue had monoallelic deletion of the 13q14 region and corresponding loss of RB1 and FOXO1A protein expression.

**Conclusions:**

This case report may strengthen the hypothesis that thymofibrolipoma is a neoplastic lesion and a variant of thymolipoma and that thymofibrolipoma and lipofibroadenoma are different names for the same lesion. The name “lipofibroadenoma” was given to the lesion because of its histological resemblance to fibroadenoma of the mammary gland. However, this name does not reflect the pathogenesis of this lesion, and the name “thymofibrolipoma” would be preferable. It will be necessary to discuss whether lipofibroadenoma should be listed as an independent entity in the WHO classification.

**Supplementary Information:**

The online version contains supplementary material available at 10.1186/s13000-022-01260-1.

## Background

Thymofibrolipoma has been described as a variant of thymolipoma. It was first reported in 2001 by Moran et al., who revealed that thymofibrolipoma was composed of extensive areas of collagenous tissue interspersed with islands of mature adipose tissue and strands of thymic tissue.[1].

## Case presentation

We encountered a case of thymofibrolipoma for which immunohistochemical and cytogenetic analyses were performed.

**Fig. 1:**
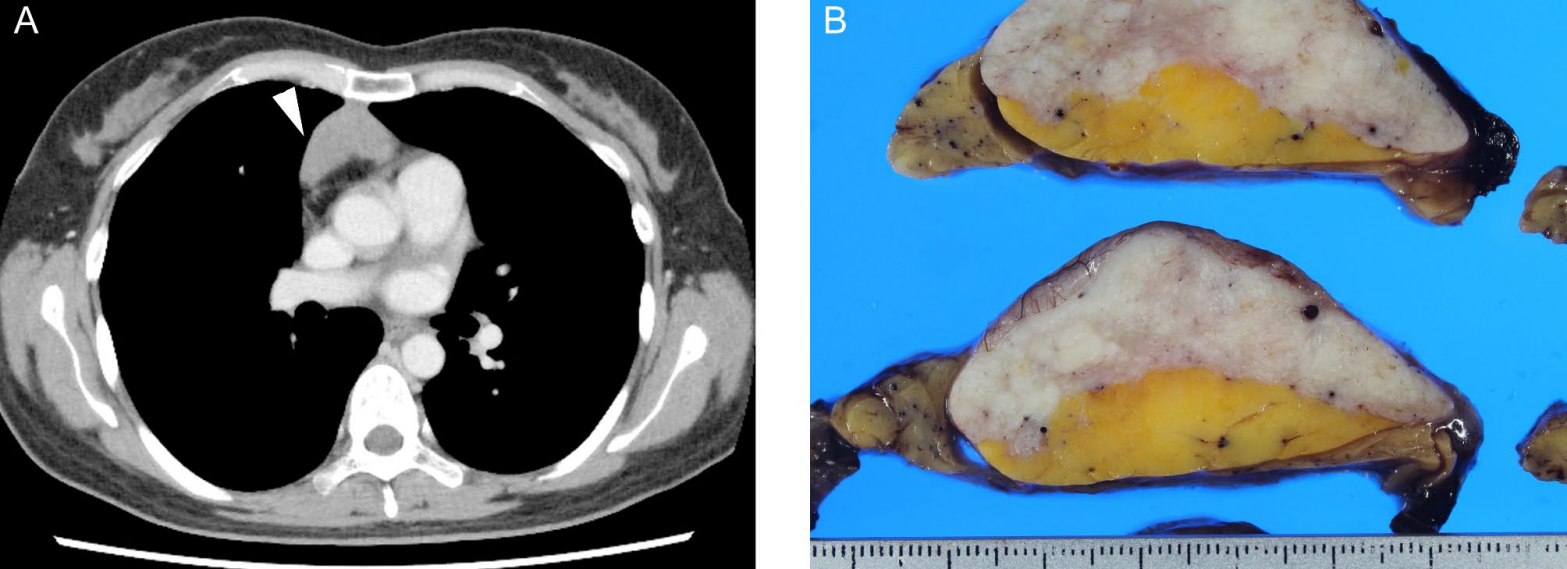
Clinical images: (A) Chest computed tomography revealed a non-contrast-enhanced mass lesion in the anterior mediastinum. (White arrowhead) Macroscopic images: (B) The cut surface of the material in the anterior mediastinal tumor showed a yellowish lipomatous component and a uniform whitish fibrous component

A 43-year-old woman had an anterior mediastinal tumor, and chest contrast-enhanced computed tomography was performed at a periodic health check-up for observation of a suspected thymic cyst. (Fig. 1 A) During follow-up, the tumor continued to grow slowly, and thoracoscopy-assisted mediastinal tumor resection was performed because a neoplastic lesion could not be ruled out.

**Fig. 2:**
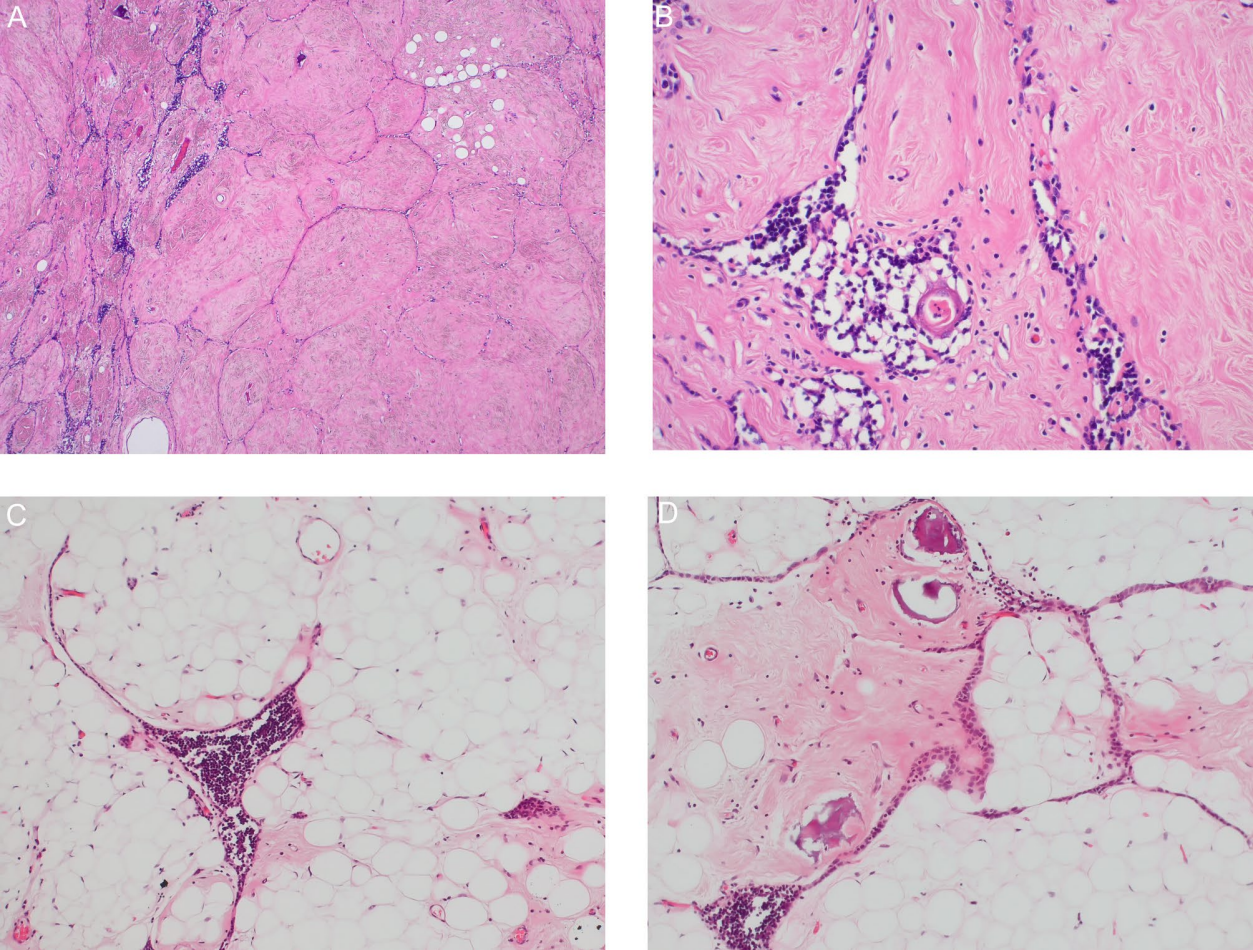
Microscopic images: Collagenous tissue accompanied by strands or islands of thymic tissue and scattered mature adipose tissue, (A) A low-magnification image (B) A high-magnification image Microscopic images: (C) Adipose tissue accompanied by strands or islands of thymic tissue. (D) Adipose tissue accompanied by collagenous tissue and scattered calcifications

Macroscopically, the tumor was a mass-like lesion that had a relatively clear border with the surrounding thymus. The cut surface of the tumor was composed of a yellowish lipomatous component and a uniform whitish fibrous component with elastic stiffness. (Fig. 1B) Microscopically, the whitish fibrous component was mainly composed of fibrous tissue with sparse spindle cells against a background of collagenous fibers. The fibrous tissue was accompanied by strands of thymic tissue, islands of adipocytes and scattered area of calcifications. (Figs. 2 A, 2B) In the yellowish lipomatous component, mature adipocytes and strands or islands of thymic tissue with small lymphocytes were observed. (Fig. 2 C) As a minor component, fibrous tissue was also observed in the lipomatous component. (Fig. 2D) Age-appropriate atrophic thymic tissue was found in the partially resected nontumor area. No neoplastic or hyperplastic lesions were found.

**Fig. 3:**
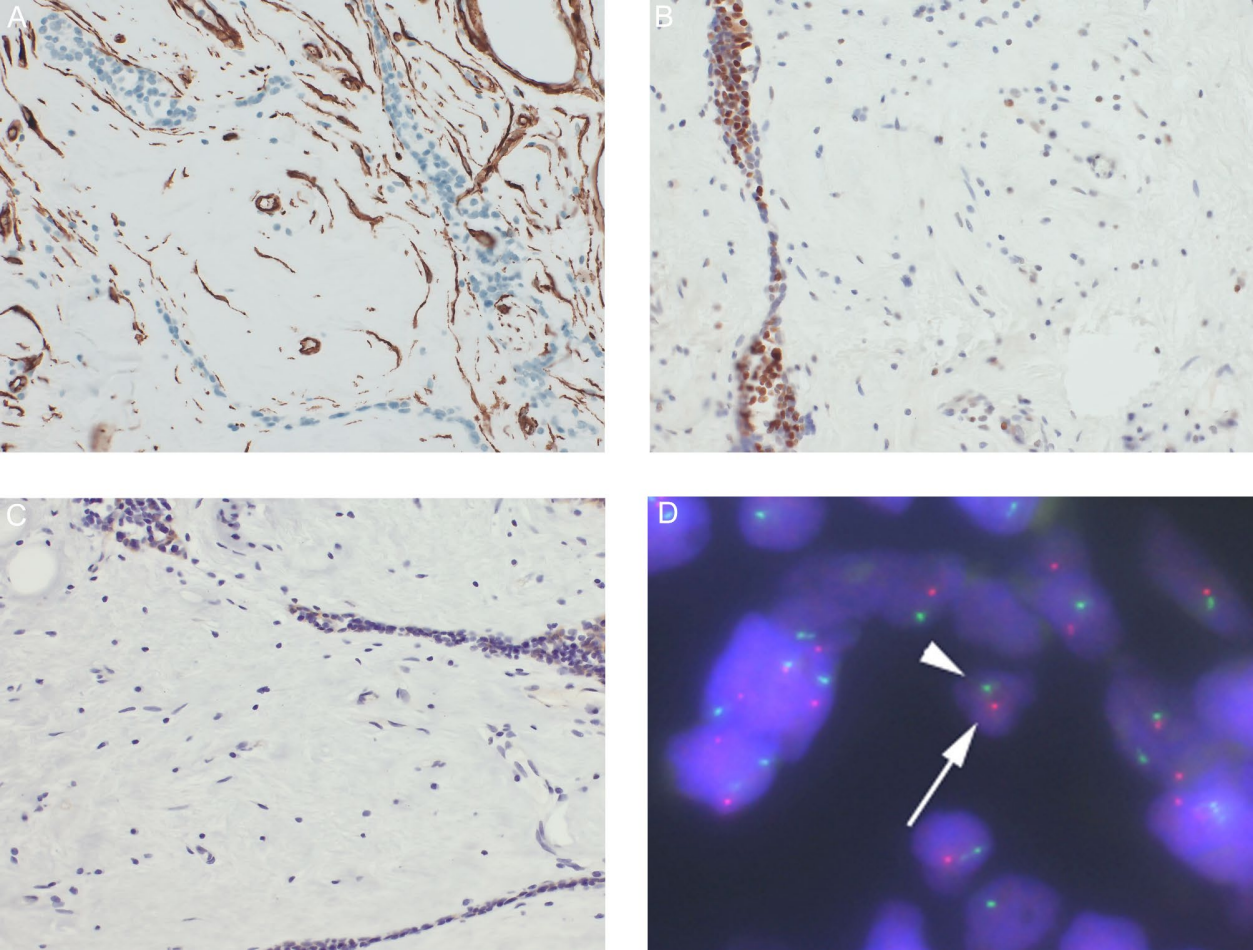
Immunohistochemical images: Spindle cells stained positive for CD34 (A) and negative for RB1 (B) and FOXOA1 (C) Fluorescence in situ hybridization assay: (D) Monoallelic loss of the 13q14 region was found. (White arrow: D13S319 probe red signal, white arrowhead: 13q34 probe green signal)

The strands or islands of thymic tissue were composed of CK19(+) thymic epithelial cells and small lymphocytes, which were predominantly immature T lymphocytes showing the following profile: CD20(-), CD3(+), CD99(+), and TdT(+). Hassall bodies were also observed in some thymic tissue. The spindle cells in the fibrous tissue showed SMA and CD34 expression and had lost RB1 and FOXO1A expression. (Figs. 3 A, 3B, 3 C) Fluorescence in situ hybridization (FISH) analysis using a probe specific for chromosome 13q14 (Vysis LSI D13s319/13q34 FISH Probe Kit, Abbott, Abbott Park, IL) detected a single signal in the tumor cells and two signals in the nuclei of normal cells, demonstrating monoallelic deletion of the 13q14 region in the tumor cells. (Fig. 3D) Based on the morphological and immunohistochemical analyses, we diagnosed the tumor as a thymofibrolipoma. The patient was discharged uneventfully. During the 8-month follow-up period, the patient did not show any evidence of recurrence or metastasis.

## Discussion and conclusions

Thymolipoma is classified in the current WHO classification as a mesenchymal tumor of the mediastinum.[2] It is a benign tumor composed of a mixture of mature adipose tissue and nonneoplastic thymic tissue. Thymofibrolipoma has been reported as a variant of thymolipoma, and to date, 3 cases have been reported.[1, 3] Thymofibrolipoma was first described in 2001 by Moran et al., who revealed that it was composed of extensive areas of collagenous tissue interspersed with islands of mature adipose tissue and strands of thymic tissue.[1] One report described chromosomal abnormalities, suggesting that thymolipoma may be a neoplastic lesion.[4] In our case, monoallelic 13q14 deletion and the corresponding loss of RB1 and FOXO1A protein expression were found. Monoallelic 13q14 deletion has been found in certain neoplasms, including spindle cell/pleomorphic lipoma, cellular angiofibroma and myofibroblastoma.[5, 6] Whether this finding is a coincidence or a recurrent feature will require further investigation, but if it is a recurrent feature, it may strengthen the hypothesis that thymofibrolipoma is a neoplastic lesion and a variant of thymolipoma.

Variants of lipoma (angiolipoma, chondroid lipoma, myolipoma, myelolipoma, etc.) differ from conventional lipoma in terms of their characteristic microscopic features.[6]　One variant of lipoma, called sclerotic lipoma, was characterized as having a prominent fibrosclerotic stromal matrix.[7] As there are many variants of soft tissue lipoma, it is not surprising that there would be many variants of thymolipoma if thymolipoma is a neoplastic lipomatous lesion. Just as sclerotic lipoma is a variant of soft tissue lipoma, thymofibrolipoma may be a variant of thymolipoma.

The current WHO classification lists lipofibroadenoma as a rare form of thymoma.[2] To date, 12 cases have been reported.[8–19] Lipofibroadenoma was first described in 2001 by Kuo et al., who revealed a histology similar to that of fibroadenoma of the mammary gland, namely, that lipofibroadenoma was composed of fibrotic and hyaline stroma interspersed with narrow strands of thymic tissue and mature adipose tissue.[8].

To date, 12 cases of lipofibroadenoma and 3 cases of thymofibrolipoma have been reported. In all reported cases, extensive areas of collagenous tissue interspersed with islands of mature adipose tissue and strands of thymic tissue are the main microscopic features. (Supplementary Tables 1, 2) Based on the descriptions of the histopathological features and microscopic figures of lipofibroadenoma and thymofibrolipoma in previous reports, it is possible that they are different names for the same lesion.

The name “lipofibroadenoma” was given to the lesion because of its histological resemblance to fibroadenoma of the mammary gland.[8] However, the thymic epithelial cells in this lesion had morphological and immunohistochemical features consistent with normal thymic components; thus, its name does not reflect the pathogenesis of this lesion. If our hypothesis that thymofibrolipoma is a neoplastic lesion and a variant of thymolipoma, described in the previous paragraph, is correct, the name “thymofibrolipoma” may be preferable.

The current WHO classification lists lipofibroadenoma as an independent entity of thymoma; however, as we have suggested in our report, if thymofibrolipoma, as a subtype of thymolipoma, and lipofibroadenoma refer to the same lesion by different names, it would be necessary to discuss whether it is appropriate to describe lipofibroadenoma as an independent entity.

## Electronic supplementary material

Below is the link to the electronic supplementary material.

Supplementary Material 1

## Data Availability

All data generated or analyzed during this study are included in this published article.

## Abbreviations

FISH: Fluorescence in situ hybridization
