# Supplementary material for: Thymofibrolipoma: a case report and review of the literature
Source: Diagn Pathol. 2022 Oct 12;17:77. doi: 10.1186/s13000-022-01260-1 (PMC9554969; doi:10.1186/s13000-022-01260-1)
Supplement: Supplementary file 1 — Supplementary Material 1 [file 13000_2022_1260_MOESM1_ESM.docx]

Supplemental Information for:

Thymofibrolipoma: A case report

Authors:

Ryu Jokoji, M.D.^1^, Emiko Tomita, M.D.^2^

^1^ Department of Pathology, Nippon Life Hospital, Osaka, Japan

^2^ Department of Thoracic Surgery, Nippon Life Hospital, Osaka, Japan

Corresponding author:

Ryu Jokoji M.D.

Department of Pathology, Nippon Life Hospital, 2-1-5 Enokojima, Nishi-ku, Osaka, Japan, 550-0006

Phone: +81-6-6443-3446, Fax: +81-6-6443-3561, e-mail: r.jokoji@gmail.com

Supplementary Table 1.

Summary of the three reported cases of thymofibrolipoma.

| Case summary  (age/sex/tumor size/coincidence) | Description of the histological features in the report | References |
| --- | --- | --- |
| 9, Female, 5 cm | “The other was composed of extensive areas of fibroconnective tissue in which areas of pseudolobulation were evident under higher magnification. Each of these pseudolobules was separated by thin strands of epithelial cells (remnants of thymic tissue). In other areas, the fibroconnective tissue and the thymic tissue merged with areas of mature fat. Extensive areas of fat were present within the fibroconnective tissue, “ | Moran^1^ |
| 32, Male, 14 cm |  |  |
| 9, Female, 9.0x7.0x7.5 cm | “The other contained areas of collagenous tissue interspersed with mature adipose tissue. Strands of epithelial cells admixed with small lymphocytes were embedded in the fibroadipose stroma.” | Kang^2^ |

* N.D.: no data

Supplementary Table 2.

Summary of the 12 reported cases of lipofibroadenoma.

| Case summary  (age/sex/tumor size/coincidence) | Description of the histological features in the report | References |
| --- | --- | --- |
| 62, Male, N.D., Pure red cell aplasia & Thymoma type B1 | “The lipofibroadenoma part of the composite tumor is composed of slender elongated epithelial strands forming various figurate structures in a loose fibrous stroma with scattered individual or groups of fat cells.” | Kuo^3^ |
| 32, Male, 3.5 cm, | “Lymphocytes were scarce within the tumor with abundant interstitial stroma, and the tumor epithelial cells displayed restiform and dendritic structures.” | Onuki^4^ |
| 56, Male, 12x7x4.5 cm, Thymoma typeB1 | “It is mainly composed of adipocytes, thymic epithelial cells, and fibrous mesenchyme. Adipocytes are scattered in a loose fibrous mesenchyme, thymic epithelium is linear, glandular duct-like, sometimes extruded into lacunae.” in Chinese | Wang^5^ |
| 23, Female, 21x7.5x7 cm, Thymoma type B1 | “The cut surface showed that the lesion was solid and fibrotic in appearance and that it had scattered areas of adipose tissue.” “Microscopic examination showed an unusual composite tumor of a conventional type B1 thymoma and a lipofibroadenoma.” | Aydin^6^ |
| 21, Male, 10x6x4 cm | “The tumor was observed with irregularly connected figurate strands of thymic epithelial cells in a fibrous tissue, in which the fat cell was distributed singly or multifocally.” | Qu^7^ |
| 20, Male, 23x14x5 cm | “Microscopic review revealed mature adipose tissue and fibrosis with narrow strands of predominantly epithelial cells with a few scattered small lymphocytes and rare Hassall corpuscles, reminiscent of thymic parenchyma.” | Makdisi^8^ |
| 55, 4.5x1.8x1.3 cm | “The lipofibroadenoma area showed narrow strands of CK19+ epithelial cells compressed by the growth of stromal component containing adipocytes, fibroblasts and collagen fibers.” | **Hamada^9^  **Kurebayashi^10^ |
| 29, Male, 6x3.5 cm | “Histologically, it was composed of adipocytes mixed with fibrous tissue and strands of epithelial cells accompanied by lymphocytes.” | Kojima^11^ |
| 21, Male, 23 cm | “Microscopic review revealed a tumour composed of thymic elements, mature adipose tissue and fibrosis. “ | Akkaya^12^ |
| 29, 5.4x2.4x6.5 cm, Thymoma type B1 | “Microscopic examination revealed a lesion comprising strands of compressed bland epithelial cells separated by fibrotic and hyalinized stroma.” “Lobules of adipose tissue, calcification, and lymphoid cells with Hassall's corpuscles were entrapped in the stroma.” | Hui^13^ |
| 28, Male, 8.8x6.7x4.2 cm | “Microscopically, the tumor was well-circumscribed, showing scattered cord-like structures composed of epithelial-like cells embedded in abundant fibrotic and hyaline stroma and multiple fat cells.” | Hakiri^14^ |
| 64, Female, 16x8x6 cm | “Microscopy revealed that the yellow, greasy part of the tumor was made of lobules of mature adipocytes. The solid area showed strands of epithelial cells within a fibrotic and hyaline stroma.” | Bolca^15^ |

* N.D.: no data

**These reports describe the same case.

Supplemental References

1. Moran CA, Zeren H, Koss MN. Thymofibrolipoma. A histologic variant of thymolipoma. *Arch. Pathol. Lab. Med.* 1994;118:281–282.

2. Kang G-H, Han J, Kim TS, et al. Thymofibrolipoma - A brief case report -. *Korean J. Pathol.* 2010;44:338.

3. Kuo T, Shih LY. Histologic types of thymoma associated with pure red cell aplasia: a study of five cases including a composite tumor of organoid thymoma associated with an unusual lipofibroadenoma. *Int. J. Surg. Pathol.* 2001;9:29–35.

4. Onuki T, Iguchi K, Inagaki M, et al. [Lipofibroadenoma of the thymus]. *Kyobu Geka* 2009;62:395–398.

5. Wang Y-L, Yi X-H, Chen G, et al. [Thymoma associated with an lipofibroadenoma: report of a case]. *Zhonghua Bing Li Xue Za Zhi* 2009;38:556–557.

6. Aydin Y, Sipal S, Celik M, et al. A rare thymoma type presenting as a giant intrathoracic tumor: lipofibroadenoma. *Eurasian J Med* 2012;44:176–178.

7. Qu G, Yu G, Zhang Q, et al. Lipofibroadenoma of the thymus: a case report. *Diagn. Pathol.* 2013;8:117.

8. Makdisi G, Roden AC, Shen KR. Successful Resection of Giant Mediastinal Lipofibroadenoma of the Thymus by Video-Assisted Thoracoscopic Surgery. *Ann. Thorac. Surg.* 2015;100:698–700.

9. Hamada K, Kaseda K, Omura S, et al. A Case of Lipofibroadenoma of the Thymus. *Japanese Journal of Lung Cancer* 2018;58:237–238.

10. Kurebayashi Y, Hayashi Y, Emoto K, et al. Lipofibroadenoma arising in hyperplastic thymic tissue: Possible perivascular origin of lipofibroadenoma. *Pathol. Int.* 2021;71:275–277.

11. Kojima I, Matsuyama T, Tateyama H, et al. A case of lipofibroadenoma of the thymus. *Pathol. and Clin. Med.* 2018;36:265–269.

12. Akkaya B, Yilmaz DB, Ozbudak IH, et al. Lipofibroadenoma of the thymus: a case report. *Virchows Arch.* 2018;473:S272.

13. Hui M, Paul TR, Uppin SG, et al. Lipofibroadenoma with B1 thymoma: A case report of a rare thymic tumor. *Indian J. Pathol. Microbiol.* 2018;61:630–632.

14. Hakiri S, Kawaguchi K, Tateyama H, et al. Thymic lipofibroadenoma accompanied with largish calcifications. *Gen. Thorac. Cardiovasc. Surg.* 2021;69:394–397.

15. Bolca C, Has A, Bobocea A, et al. A Rare Thymic Tumor - Lipofibroadenoma - Always a Postoperative Surprise. *In Vivo* 2021;35:3623–3626.
